# From Dark to Light – An Overview of Over 70 Years of Endocrine Disruption Research on Marine Mollusks

**DOI:** 10.3389/fendo.2022.903575

**Published:** 2022-07-07

**Authors:** István Fodor, Zsolt Pirger

**Affiliations:** Ecophysiological and Environmental Toxicological Research Group, Balaton Limnological Research Institute, Eötvös Loránd Research Network (ELKH), Tihany, Hungary

**Keywords:** invertebrate, marine mollusk, endocrine disruption, sex steroids, historical trends

## Introduction

The major sex steroids in humans, progesterone (P), testosterone (T), and 17β-estradiol (E_2_), were discovered in the 1920-30s (1–3). Not long afterwards, the presence of vertebrate sex steroids in marine molluscan tissues was first reported (4). At the time, it was reasonable to assume that these steroids were of endogenous origin in mollusks (and in invertebrates in general) and were possibly used as hormones in the same way as they are in vertebrates. This led people to investigate the effects of sex steroids in mollusks, the first study was published as early as 1959 (5). All but a handful of the 300+ scientific papers and reviews, mainly using analytical, immunological, molecular, and behavioral measurements, have since concluded either that mollusks are able to biosynthesize vertebrate steroids *de novo*, appear to contain steroid receptor-like binding activity, or respond in one way or another when exposed to vertebrate steroids.

The research area had two key discoveries that served as driving forces. First, the notorious discovery that the anti-fouling compound tributyltin (TBT) is the main causative agent of penis growth in the females of marine snails (6). Due to the previously described presence of T in molluscan tissues, this ‘androgenic’ effect led to the hypothesis that the phenomenon was caused by alteration of T production or metabolism by TBT [reviewed by (7, 8)]. Second, that hormone residues of human origin are present in the environment including surface waters (9) and that estrogenically active compounds emitted by wastewater treatment plants induced massive vitellogenin production in immature fish (10). Because of the worldwide concern about the hormone residues (and other compounds) as possible endocrine disruptors (EDs) in the aquatic environment, most of the research has focused on the effects of estrogens and androgens on reproduction on fish because the hormones and their effects are so similar between the two taxonomic groups. Some robust and relatively accurate test procedures, using fish, exist for detecting and quantifying the biological activity of estrogenic and androgenic activity in water. The drawback to these tests is that they are very expensive and, because fish are vertebrates, require a lot of paperwork in relation to vivisection laws in developed countries. This and the concern about the possible endocrine disruption of mollusks, which also have an important ecological and economic role, led to a burst of activity in the 1990s and 2000s to use them as alternative test animals for monitoring vertebrate-type EDs.

Starting from 2010, critical reviews called into doubt the claims that reproductively-related peptides, sex steroids, and their receptors that are present in vertebrates, and that can also be found in mollusks, necessarily have the same function (8, 11–18). These efforts pointed out many weaknesses of different techniques and approaches (e.g., immunoassays, biomarkers, nominative determinism) used over 70 years in this area. This opinion paper deals with the historical trends of endocrine disruption research in marine mollusks and presents the way to the recognition that invertebrate endocrinology differs from the well-characterized vertebrate endocrine system. We will focus on the sex steroids and not discuss compounds that are not themselves steroids but, for example, act as sex steroids (e.g., bisphenol A) or modify the actions of sex steroids (e.g., cyproterone acetate) in vertebrates. We will also discuss some underexplored research lines (e.g., membrane receptors) and delineate some possible future directions that can throw light on the molluscan endocrinology.

## Historical Trends of ED Research in Marine Mollusks

### Analytical Measurements and Immunoassays Revealed That Vertebrate Sex Steroids Occur in Molluscan Tissues

When vertebrate sex steroids in marine molluscan tissues were first reported, nothing was known about the reproduction-mediating endocrine system of mollusks. Building on the pioneer finding, numerous (>50) subsequent publications, mainly using analytical methods (e.g., gas/liquid chromatography coupled mass spectrometry) and immunoassays (e.g., enzyme/radioimmunoassay), have reported the presence of different vertebrate sex steroids in a wide range of (marine) mollusks [reviewed by (11, 14)]. Some (but, it must be stressed, not all) studies found that steroid concentrations sometimes 1) show seasonal changes, 2) are different between tissues and sexes, and 3) can alter due to other contaminants (11). These findings have encouraged people to believe mollusks have a vertebrate-type endocrine system and are able to biosynthesize vertebrate steroids *de novo*. As a result, researchers have extensively investigated the effects of sex steroids in (marine) molluscan species over the last 60 years [reviewed by (8)].

### Proteins Associated With *De Novo* Synthesis and Receptor-Mediation of Vertebrate Sex Steroids Are Present in Marine Mollusks

**Figure 1:**
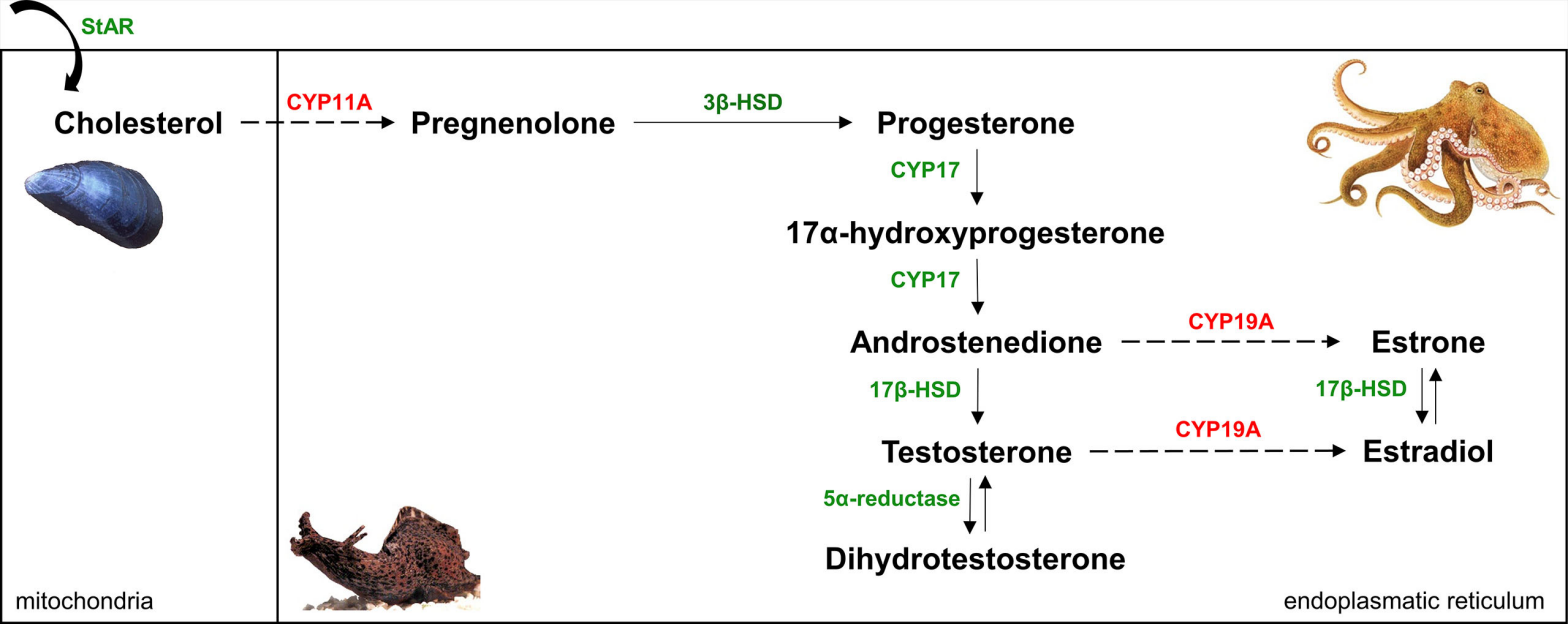
Simplified (‘delta-5-steroids’ formed from pregnenolone are not shown) vertebrate sex steroidogenesis pathway [modified after (19)]. Six reactions (solid arrows), though some with low activity, and the enzymes that catalyze (indicated by green color) them appear to be present in mollusks. CYP11A and CYP19A (marked by red color) have not been found in molluscan genomes so far, this supports the notion that two crucial steps - cholesterol side-chain cleavage and aromatization (dashed arrows) - of the classical vertebrate steroid biosynthetic pathway are absent in mollusks.

Besides measuring sex steroid levels in marine mollusks, researchers have been interested in proving the presence of proteins involved in the synthesis and receptor-mediation of P, T, and E_2_. The vertebrate sex steroids are formed by a process starting with the side-chain cleavage of cholesterol and finishing with the aromatization of T to E_2_ (13). Six steps of the multistep pathway (though some with low activity) appear to be present in marine mollusks with the genes encoding the relevant catalyzing enzymes also found in molluscan genomes (**Figure 1**) (11, 13, 14). The evidence for their occurrence in marine mollusks is strong – e.g., conversion of pregnenolone to P by the common octopus (*Octopus vulgaris*), common cuttlefish (*Sepia officinalis*), atlantic sea scallop (*Placopecten magellanicus*), and blue mussel (*Mytilus edulis*) (20–23); conversion of androstenedione (Ad) to T by eastern oyster (*Crassostrea virginica*) and *S. officinalis* (22, 24); conversion of estrone to E_2_ by *C. virginica* and *M. edulis* (24, 25); and conversion of T to dihydrotestosterone by *M. edulis* (26). The question whether the two remaining crucial steps, cholesterol side-change cleavage and aromatization (catalyzed by CYP11A and CYP19A, respectively), are present in marine mollusks is of substantial interest in this area. To the best of our knowledge, there is no firm evidence that any metazoans other than vertebrates can carry out cholesterol side-chain cleavage (27). In contrast, previous studies have demonstrated either aromatase activity (22, 28–32) or immunostaining of cells with antibody raised against human aromatase (29, 33, 34) in marine mollusks.

The discovery of molluscan nER, a homologue of vertebrate nER, in marine mollusks in the early 2000s confirmed the notion that these species have a vertebrate-type endocrine system and further increased the activity to develop mollusks as test animals for endocrine disruption. The first molluscan nER was biochemically and immunohistochemically identified in *O. vulgaris* (35), then the first cDNA sequence was identified the following year in the sea hare (*Aplysia californica*) (36). To note, the authors of the latter paper concluded that nER became independent of hormone regulation in the *Aplysia* lineage.

### Nominative Determinism

Nominative determinism is, in our opinion, a major problem in invertebrate research. Simply put, there are protein/DNA sequences in invertebrates that are homologous to hormones, receptors, and enzymes in vertebrates. These proteins are invariably given the names that describe their function in vertebrates and seduce people into concluding ‘if that protein has name and function x in humans, then it must also have function x in the invertebrate species that I am working on’.

An example is the case of the invertebrate GnRH/corazonin (invGnRH/CRZ) neuropeptides. The original term invGnRH is a misnomer derived from the misinterpretation of sequence and function of the first discovered invGnRH/CRZ in *O. vulgaris* (37). At the time, this further strengthened the assumption that invertebrate and vertebrate endocrinology are similar including the presence of a homologous system for the vertebrate hypothalamic-pituitary-gonadal axis. Since then we have found that these peptides are multifunctional and do not have a specific reproductive function as seen in vertebrates (38–43). As a result, more studies have investigated these peptides and/or their assumed related molecules [e.g., follicle-stimulating hormone (FSH)] in mollusks with antibodies raised against vertebrate molecules (44–47); most likely resulted in non-specific results and so inappropriate conclusions.

Another example is the abovementioned, inappropriately named ‘molluscan nER’ which, study after study has shown, does not bind to estrogens at all (36, 48–55). And yet this fact has not stopped people trying to prove that it can be used as a biomarker for estrogen exposure in mollusks. A key driver of this interest was a study published in 2010 by Ciocan and her co-workers that showed some very large changes in mRNA expression of the gene for the nER when *M. edulis* was exposed to a variety of estrogens (56). Several other studies have also measured nER expression in mollusks with getting highly variable results: some found positive changes, some found negative changes, and some found no changes [reviewed by (13)]. We think one of the biggest problems in the field is the positive publication bias: researchers tend to quote the exciting ‘positive’ studies in their Introductions and Discussions and the studies showing ‘no effect’ or a ‘negative effect’ do net get quoted. Despite these facts and anomalies, people still like measuring the mRNA levels of nER in mollusks and claim that it is linked to stage of the reproductive cycle or is upregulated by estrogen treatment.

### On the Way to the Light: Uptake and Esterification of Steroids by Mollusks

The most common type of study of this research area involvessampling animals at different reproductive stages, killing them and homogenizing their tissues homogenized and then measuring the levels of sex steroids using commercial immunoassay kits. If, as it often happens, these measurements are different between one reproductive stage and another, the authors claim that this is ‘evidence’ of an involvement of vertebrate steroids in the control of reproduction in mollusks. A critical review by Scott pointed out that people who have published such studies have in most cases completely ignored the facts that 1) the environment (including the laboratories) is awash with vertebrate steroids; 2) (marine) mollusks have a remarkable ability to absorb the common human sex steroids from water; and 3) they have an even more remarkable ability to store these steroids and/or their metabolites long-term in the form of fatty acid esters (12). Since no live animal studies have been carried out under immaculate steroid-free conditions (humans are a substantial potential source of contamination in laboratories) (11), it is impossible to know for sure whether a steroid level has changed because that is what it should have done if it really was a hormone, or because there was increased contamination, or a reduction in esterification, or an increase in de-esterification [or at least fourteen other potential mechanisms that could produce the same results (12)]. In fact, uptake and esterification of steroids by marine mollusks were firmly demonstrated in 2001 (57) and have since been confirmed many times by investigating different marine molluscan species (26, 31, 58–62). Yet publications that make no mention of these mechanisms continue to appear in the literature. It is hard to know whether the authors just do not read any of the relevant papers or reviews, know about it, but do not appreciate its significance, or deliberately choose not to mention it because it is inconvenient to their narrative.

### Spread of High-Throughput Sequencing Points Out the Lack of Key Sequences

Findings on sex steroid uptake by marine mollusks raised the idea that it is not possible to conclude that just because steroids are present in molluscan tissues, they are endogenously synthetized. The spread of next-generation sequencing techniques allowed researchers to sequence the genome and multiple transcriptomes of molluscan species and to identify their protein and receptor repertoires related to sex steroids. Such studies clearly demonstrated that any homologous sequence to vertebrate genes coding for CYP11A and CYP19A is not present in (marine) mollusks (**Figure 1**) (19, 63–65). In fact, outside of vertebrates, CYP19A has so far been found only in cephalochordates (66–68). Although CYP11A was previously identified in amphioxus *Branchiostoma belcheri* (66), it was later shown not to be a CYP11A but a distant paralog (69). The current theory is that the appearance of CYP11A is a result of a vertebrate-specific gene duplication (27, 69). To fill in the gaps in the pathway, two P450 enzymes that are known to be present in mollusks, CYP10 and CYP3A, were suggested to fill in the roles of CYP11A and CYP19A, respectively (70). However, it must be stressed that, at the moment, this is pure speculation. Comparative genomic and transcriptomic studies also presented that there is zero evidence that the gene of nuclear androgen receptor and nuclear progesterone receptor is found in molluscan genomes, supporting the idea that these genes did not evolve until late in vertebrate evolution (27).

These findings question the claims of the abovementioned studies that, for example, aromatase activity and CYP19A are present in mollusks. In fact, the methodical approaches, such as using labelled Ad and immunostaining with vertebrate antibodies, of those studies have since been questioned (11, 13). Immunostaining has especially been deemed to be highly unreliable in such investigations. For example, despite the gene for CYP19A being not present in mollusks, antibodies against human aromatase show positive immunostaining in these species. Because of the technical problems such as cross reactivity, variability, and wrong application (71, 72), one has to be careful when making immunostaining with antibodies (especially polyclonal ones) against mammalian proteins for identifying or localizing specific proteins in invertebrate tissues.

Finally, reference must be made that the robustness of the bioassay data in most of the papers which claim that mollusks use sex steroids as hormones in the same way as vertebrates, or seem affected by sex steroids, has been questioned (e.g., single experiments only, lack of replication, questionable endpoints, low effect sizes, lack of dose-responsiveness) (8). Highlighting two iconic examples: in contrast to the initial findings/conclusions felt to be encouraging, 1) TBT was clearly demonstrated to exert its effect not *via* T but the retinoic X receptor (73–77) and 2) estrogens were shown to have absolutely no effect on vitellogenin production in bivalves (78–82). In his critical evaluation, Scott indicated the importance of a monotonic dose–response relationship for demonstrating a specific ligand-dependent effect and presented its lack in most of the steroid-effect relationships investigated in mollusks (8). However, since there are numerous examples of non-monotonic curves (U-shaped, inverted U-shaped, flat, irregular) in research papers in ecotoxicology [reviewed by (83)], the traditional concept of dose-responsiveness has been reconsidered in endocrine disruption research in the last decade. We do not exclude the possibility of genuine non-monotonic curves but would like to point out that the underlying mechanisms should be determined in each case to add something really informative to the field.

### Outstanding Questions and Possible Future Directions

We think one can agree that knowledge about molluscan endocrinology is ‘in the dark’, however, the introduction of some underexplored research lines to the discussion can contribute to throwing light on it.

Because of the worldwide concern about the possible endocrine disruption caused by hormone residues in surface waters, recent reviews, although they agree that the definition of sex steroids as ‘hormones’ cannot be applied in mollusks as in vertebrates, tried to fill in the gaps in the steroid signaling and raised the idea of membrane receptors/non-genomic pathways to explain the assumed physiological effects of sex steroids [reviewed by (18, 84)]. The rapid (within seconds to minutes) effects of sex steroids *via* membrane receptors is, though not yet completely understood, well-documented in vertebrates (18); for example, the effects of E_2_ *via* different membrane estrogen receptors (mER) and the effects of P *via* the five membrane progesterone receptors (mPR) are well-known (85, 86). In mollusks, most of the effort to prove this possible mode of action were performed by the investigation of hemocytes of *Mytilus* spp [reviewed by (18)]. These works demonstrated that E_2_ rapidly induced lysosomal membrane instability, production of intracellular reactive oxygen species, lysozyme release and nitrogen-oxide production in *Mytilus* hemocytes; the underlying mechanisms, similar to those identified in mammalian cells, involved cytosolic Ca^2+^ increase, activation of MAPKs and PKC, and phosphorylation of transcription factors (e.g., CREB) (87–90). Without judging the robustness of these findings, we would like to mention that only the lysosomal membrane stability-decreasing effect has so far been properly verified and this, as the authors also pointed out, is a classic ‘immune response’ rather than an ‘endocrine response’ (i.e. not a proof that E_2_ acts as a hormone in mussel hemocytes) (18). At the moment, it is unknown whether E_2_ binds to a specific membrane receptor in mollusks, to a receptor for other compound(s) (cross-reactivity), or if there is a receptor involved at all.

Based on the assumptions that at least one functional mER is present in mollusks and that the upregulation of nER mRNA is reflected at the protein level, Tran and her co-workers proposed a mechanistic model that considers an interplay between non-genomic and genomic pathways to explain E_2_ signaling in mollusks (84). Accordingly, binding of estrogens to mER(s) triggers the activation of second messengers and protein kinase cascades resulting in the phosphorylation of nER and other transcription factors. These will bind to the ERE region of the nER gene and other genes (e.g., vitellogenin). As a self-exciting process, the increased nER transcription leads to higher levels of the ER protein. In addition to stressing that, at the moment, this model is pure speculation, we would like to mention two concerns. First, one must question how such an indirect system would evolve by natural selection. Second, as presented in the 2.3. subsection, the up-regulation of nER mRNA expression in mollusks exposed to estrogens was reported only in some papers, while other papers presented down-regulation or no change. Besides mERs, a short reference must also be made on mPRs. The long-term concept was that mPRs were present only in chordates (91). However, a recent comprehensive study has revealed that although mollusks do not have a homolog to mPRα, which is responsible for mediating the most mPR functions in vertebrate cells, they have homologs to mPRβ and mPRγ (86). Importantly, the progesterone-binding ability of these proteins has not been tested and their presence does not necessarily mean the same function as in vertebrates. However, we believe that identification and deorphanization of potential mER homologs in mollusks, as well as deorphanization of these putative mPRs, could contribute to the clarification of existence of molluscan GPCRs which are responsive to vertebrate sex steroids.

Although cephalochordate ERs are also insensitive to estrogen, functional assays showed that annelid ERs bind estrogen with high affinity (92). This led to the theory that the ancestral state was estrogen-sensitive (92, 93). Here, we would like to stress that there is no more evidence for endogenous synthesis of estrogens in annelids than in mollusks. The allosteric switch of the ligand-binding domain of ‘molluscan nER’ has been proposed to become stuck in the agonist position leading to constitutive transcription/ligand-independence (92–94), and, indeed, the investigation of *C. gigas* ER supported this idea by convincingly demonstrating the vestigialization of the receptor (95). Recent findings by Markov and his co-workers suggested that an aromatized sterol (i.e. a cholesterol metabolite that have not undergone side-chain cleavage), paraestrol A, behaved as an activator of the ancestral steroid receptor (27). We agree that the term ‘estrogen-sensitive ancestral state’ has no sense outside organisms having estrogens, so vertebrates for the moment. Although we do not think that paraestrols are the ‘missing estrogens’ in mollusks, the discussion of sterols as possible hormones in mollusks can inspire the launch of new research lines and can throw light on the molluscan endocrinology, though not necessarily in relation to reproduction. Since aromatized long-chained steroids have been firmly identified in sponges and cnidarians (96, 97), we suppose that a paralogous CYP may catalyze the aromatization of sterols in mollusks. In fact, several sterols with non-reproductive properties have already been identified in mollusks (98). Their potential role is supported by a recent finding that inhibition of 5α-reductase, that is an ancient enzyme and known to use sterols (as well as T and Ad) as substrates, caused marked malformations in shell morphology during the development of two freshwater gastropods (99). Furthermore, we suppose that existence of further enzymes in mollusks that are not vertebrate orthologues but can carry steroid metabolism. When Schwarz and her co-workers exposed M. edulis to tritiated T, a considerable 22% production of tritiated water was detected. (26). There was no evidence of E_2_ or E_1_ production, however, there were plenty of unidentified peaks of radioactive organic compounds in the water. Even if this does not mean steroids have a functional role in mollusks, it supports the existence of, still unknown, pathway(s) of androgen metabolism.

## Discussion

Ever since the discovery of vertebrate sex steroids in (marine) mollusks in the late 1950s, the investigation of their presence in molluscan tissues and their effects on these species has been of substantial interest. In our opinion, there are salutary lessons to be learned from the historical trends of this research area. In the early decades, when several studies have been published showing the presence of sex steroids (and proteins involved in their synthesis and receptor-mediation) in molluscan tissues, the lack of sequence data highly influenced the area while robbing researchers of implementing appropriate investigations. Actually, one could rightly assume that mollusks have a steroid biosynthetic pathway which is equivalent to that of vertebrates. From 2000, steroid uptake experiments demonstrated that these compounds are so readily absorbed that their presence is not necessarily evidence of endogenous synthesis. Technical developments in the genomic era have also improved the area with pointing out the lack of key sequences. However, all genes in the databases of invertebrate genomes are assigned gene ontology categories of vertebrate genes, this has also exacerbated the problem of nominative determinism (12, 18). Of course, there are also many positive lessons. For example, findings in the research area showed that there are nuclear receptors (e.g., retinoid X receptor, retinoid acid receptor) that participate in endocrine processes of mollusks and other invertebrates [reviewed by (94, 100)]. These results also highlighted the power of comparative genomics when supported by *in vitro* experiments. In the post-genomic era, the combination of both functional assays and cutting-edge technology (transcriptomics, proteomics, and metabolomics) can give novel insights into the molluscan endocrinology (101). Metabolomics can especially represent an excellent tool for such investigations (102), although one has to treat the results with caution.

We believe that the research area could be improved significantly by considering some principles. First, researchers, especially newcomers, should read widely and be hypercritical of what they read and of the claims people make in their papers (i.e. selection of appropriate or adequate papers). Second, we should incorporate greater involvement of the high-throughput sequencing platforms (e.g., Illumina, Oxford Nanopore), that are reproducible and now relatively cheap, in molluscan research to generate more genomic and transcriptomic data. This would reveal the presence or lack of given sequences and help prevent experiments that make no sense (e.g., measuring the changes of FSH level in mollusks) from being conducted and published. Also, the sequence data would give the opportunity to generate species-specific antibodies to the initial morphological studies before the functional ones. Third, we would like to emphasize that the presence of a gene sequence, even if orthologue from another gene whose function has been well-characterized, is not an indication of a conserved function. Inferring the function of a protein through only homology searches without any further functional investigations can be highly misleading. Last, it is very important to design the intended experiments appropriately [e.g., relevant endpoints for the compound(s) to be tested, within-study repetition, avoiding bias].

We would like to stress that none of the opinions expressed in this paper are intended as personal criticisms of any individual or organization. Our aim is to inspire invertebrate researchers to be hypercritical and to support the contention that molluscan endocrinology differs from the well-characterized vertebrate endocrine system.

## Author Contributions

IF: Conceptualization, Writing - original draft; ZP: Writing - review & editing, Supervision. All authors contributed to the article and approved the submitted version.

## Funding

This work was supported by the National Brain Project (#2017-1.2.1 NKP-2017-00002; Z.P.), 'Hungarian Scientific Research Fund (#138039; ZP), Bolyai Foundation (#BO/00646/21/8; ZP), László János Doctoral Scholarship (#498/2021/PTE DOK; IF), New National Excellence Program of the Ministry for Innovation and Technology from the source of the National Research, Development and Innovation Fund (ÚNKP-20-3-II-PTE-888; IF), Cooperative Doctoral Programme for Doctoral Scholarships of the Ministry for Innovation and Technology from the source of the National Research, Development and Innovation Fund (KDP-2020-1018493; IF), and Scholarship for National Young Talents (NTP-NFTÖ-21-B-0212; IF).

## Conflict of Interest

The authors declare that the research was conducted in the absence of any commercial or financial relationships that could be construed as a potential conflict of interest.

## Publisher’s Note

All claims expressed in this article are solely those of the authors and do not necessarily represent those of their affiliated organizations, or those of the publisher, the editors and the reviewers. Any product that may be evaluated in this article, or claim that may be made by its manufacturer, is not guaranteed or endorsed by the publisher.
